# Surgical treatment outcomes of elastofibroma dorsi in 16 patients with 22 lesions

**DOI:** 10.1186/s12893-026-03675-9

**Published:** 2026-03-19

**Authors:** Ahmet Nadir Aydemir, Argün Kış, Mert Bektaş

**Affiliations:** 1 Faculty of Medicine, Department of Orthopedics and Traumatology, Pamukkale University, Denizli, Türkiye; 2 Faculty of Medicine, Department of Thoracic Surgery, Pamukkale University, Denizli, Türkiye; 3 Clinic of Orthopedics and Traumatology, Turgutlu State Hospital, Manisa, Türkiye

**Keywords:** Elastofibroma dorsi, Soft tissue tumor, Seroma, lesion

## Abstract

**Background:**

Elastofibroma dorsi is a rare, slow-growing, benign soft tissue tumor typically located under the scapula. Although elastofibroma dorsi is a benign lesion, it may cause symptoms such as pain and restriction of movement, and its mass-like appearance can lead to considerable patient anxiety. We aimed to present patients diagnosed with elastofibroma dorsi who had clinical complaints and underwent surgical treatment, in conjunction with the literature in this study.

**Methods:**

Our study retrospectively analyzed lesions surgically treated in the Orthopedics and Traumatology and Thoracic Surgery Clinics over the past five years, which were pathologically confirmed as elastofibroma dorsi. All patients were operated on in the prone position under general anesthesia, and all excised lesions underwent postoperative pathological examination.

**Results:**

A total of 22 elastofibroma dorsi lesions were identified in 16 patients, including 6 patients with bilateral involvement. The mean age of the patients was 62.3 years (range, 46–76). Of these lesions, 13 (59.1%) were located on the right side and 9 (40.9%) on the left. The predominant presenting symptoms were pain, limitation of shoulder motion, cosmetic concerns, and the presence of a palpable mass. Lesion sizes ranged from 5 to 10 cm in diameter. Magnetic resonance imaging or computed tomography consistently demonstrated well-defined tumors situated posterolaterally in the chest wall, between the serratus anterior and latissimus dorsi muscles. All patients underwent marginal resection following anatomical dissection of the involved muscle groups. Postoperatively, seromas developed in 8 lesions (36.4%) and a hematoma in 1 lesion (4.5%). The seromas were successfully managed with aspiration and compression bandaging, while the hematoma required surgical reintervention.

**Conclusions:**

Elastofibroma dorsi is a slowly growing benign lesion typically located beneath the inferior scapula. In symptomatic patients, surgical excision results in a marked improvement of symptoms. The most common surgical complication is the development of postoperative seroma.

## Background

Elastofibroma dorsi is a rare benign soft tissue tumor characterized by the proliferation of non-encapsulated fibrous tissue, typically located in the subscapular region [1]. While it is most commonly found between the latissimus dorsi and serratus anterior muscles in the subscapular region, it has also been reported to occur in the deltoid, axilla, olecranon, and ischial regions [1–2]. It is more frequently observed in women in their 4th to 6th decades [3]. Although it typically presents unilaterally, approximately 10% of cases can be bilateral [4]. Elastofibroma dorsi was first described by Jarvi and Saxen at the 12th Scandinavian Pathology Congress in 1959, and their studies were subsequently published in 1961 [5].

The lesion is typically slow-growing and asymptomatic, but it may cause symptoms such as swelling, functional limitations, intermittent pain, and a palpable mass with crepitus during shoulder movement [6–7]. Changes in the subscapular region and repeated microtrauma are believed to predispose to the formation of elastofibroma [8]. Magnetic resonance imaging is the most reliable and non-invasive technique for depicting the mass’s characteristic fibrous and fatty components. A biopsy is considered necessary when the lesion rapidly grows over a few months or when imaging findings are atypical [9]. Surgical treatment of elastofibroma dorsi is performed only in painful cases or when the diagnosis is in doubt. It involves complete surgical excision of the mass along with curative marginal resection [10]. Asymptomatic lesions may be managed with clinical observation; however, some authors suggest that surgery is necessary for lesions larger than 5 centimeters (cm) [11–12]. In this study, we aimed to present patients diagnosed with elastofibroma dorsi who presented with clinical complaints and underwent surgical treatment, in conjunction with the existing literature.

## Methods

**Fig. 1:**
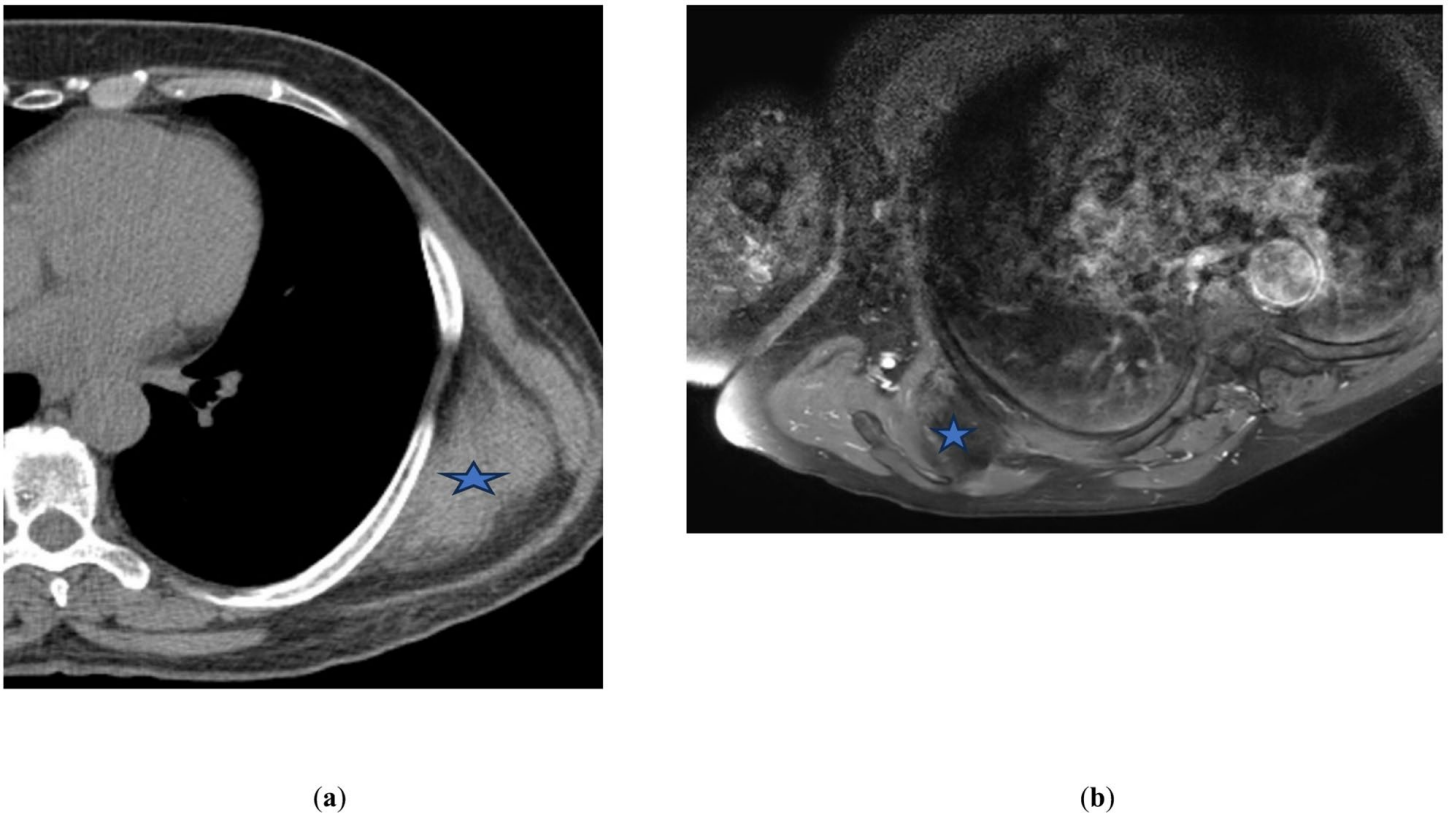
Radiological appearance of elastofibroma dorsi. **a** Axial computed tomography scan demonstrating a poorly circumscribed soft tissue mass in the subscapular region with alternating areas of soft tissue and fat attenuation (blue asterisk). **b** Axial T1-weighted magnetic resonance image showing a heterogeneous lesion with characteristic interlacing fibrous and fatty components, consistent with elastofibroma dorsi (blue asterisk)

This retrospective study reviewed 22 elastofibroma dorsi lesions identified in 16 patients who presented to the Orthopedics and Traumatology Clinic and the Thoracic Surgery Clinic of a university hospital between June 2020 and May 2025. Our study was initiated after obtaining ethics committee approval and completed within the time frame specified by the committee. Magnetic resonance (MR), computed tomography (CT), or ultrasonographic imaging in all patients demonstrated that the tumors had regular borders and were located posterolaterally in the chest wall, just beneath the serratus anterior and latissimus dorsi muscles (Fig. 1). The masses appeared isodense with skeletal muscle tissue on T1- and T2-weighted sequences, and they were seen as lesions with weak boundaries and no capsules on the MR images of the patients.

Preoperative biopsy was not performed routinely and was limited to a small number of patients in whom the lesion was located in a typical anatomical region suggestive of elastofibroma dorsi, but imaging findings were not entirely characteristic. In these selected cases, image-guided tru-cut biopsy was performed by interventional radiology to clarify the diagnosis and exclude alternative pathologies. The main differential diagnoses included other benign soft tissue tumors and, less frequently, soft tissue sarcoma. Imaging features that prompted biopsy included atypical signal characteristics or the absence of the typical fibrous–fatty pattern of elastofibroma dorsi. In addition, the lack of classic clinical symptoms in some patients contributed to diagnostic uncertainty. During the study period, two patients underwent tru-cut biopsy, which revealed other benign soft tissue tumors; these cases were therefore excluded from the final study cohort.

**Fig. 2:**
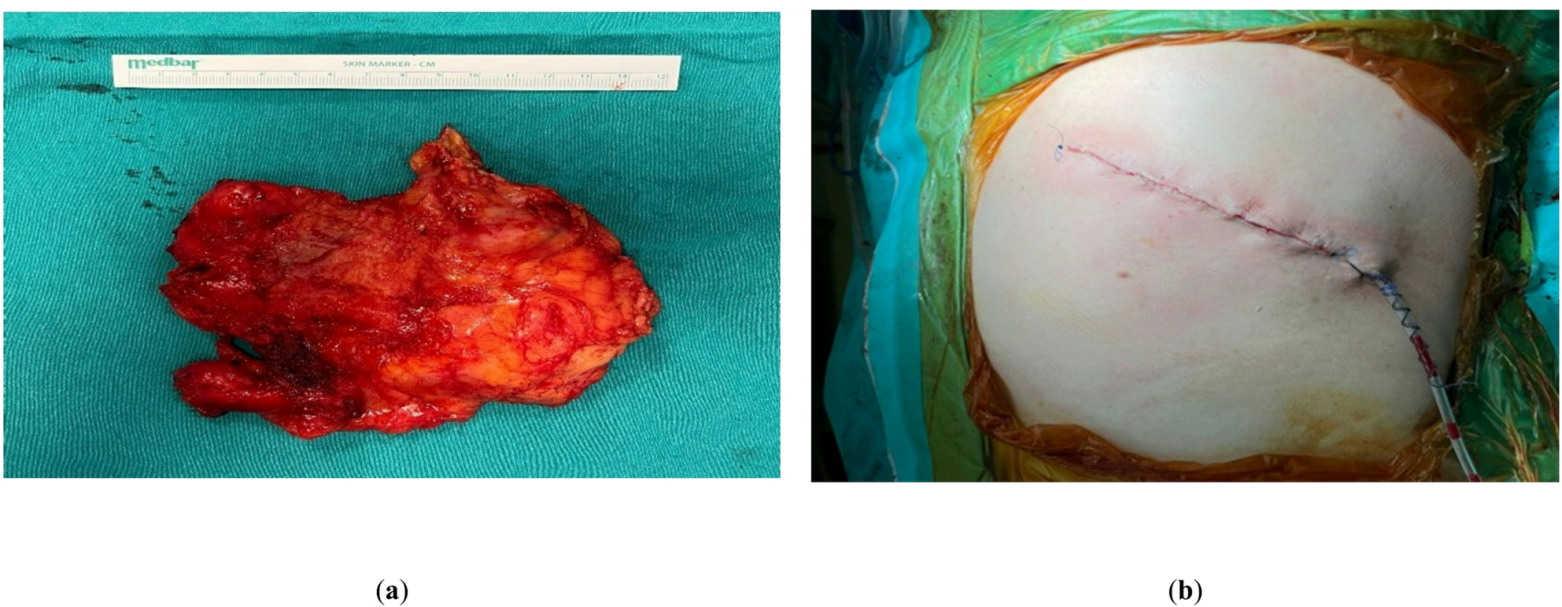
**a** Gross appearance of the excised elastofibroma dorsi specimen demonstrating a poorly circumscribed, firm mass with a reddish–yellow surface and a heterogeneous, fibrous internal structure characteristic of elastofibroma. **b** Postoperative view showing the closed surgical incision with the Hemovac drain positioned at the distal end of the incision

A transverse or oblique incision was used in all patients in the prone position at the lower pole of the scapula. Marginal resection was performed by reaching the masses after anatomical dissection of the muscle groups. It was observed that the masses did not infiltrate the chest wall or cause destruction of the underlying bone. The masses were found to consist of a macroscopic fibrous tissue in association with fatty tissue. All masses were sent to the pathology laboratory for histopathological examination (Fig. 2a). After ensuring hemostasis at the incision site, closure was performed in accordance with standard techniques and using one Hemovac drain for all patients (Fig. 2b).

## Results

In our study, the female-to-male lesion ratio was approximately 2.1:1 (15 lesions in females vs. 7 lesions in males). Of the 22 lesions, 13 (59.1%) were located on the right side and 9 (40.9%) on the left side. The mean age of the patients was 62.4 years (range, 46–76). All patients were followed for a mean duration of 19 months (range, 12–54 months) after surgery. Table 1 presents patients’ age, gender, tumor localization and size, dominant symptom, duration of symptoms before surgery, postoperative hospitalization period, and complications. Although the main presenting symptoms are summarized in Table 1, all patients expressed concern about an unidentified mass.

**Table 1:** Demographic and clinical characteristics of patients, including age, gender, tumor localization, tumor size, postoperative length of stay, and complications

Patient	Age	Gender	Side	Tumor size (cm)	LOS^1^ (Days)	Complication
1	57	Female	Right	8	4	None
2	69	Female	Right	7	3	None
3	69	Female	Left	6	3	None
4	69	Male	Left	6	2	None
5	64	Female	Right	9	6	Seroma
6	60	Female	Right	10	8	Hematoma
7	61	Female	Right	7	3	None
8	54	Female	Left	8	4	None
9	61	Male	Right	5	4	None
10	61	Male	Left	5	4	None
11	59	Female	Right	6	3	None
12	46	Male	Right	5	2	None
13	46	Male	Right	6	3	None
14	67	Female	Right	7	5	Seroma
15	67	Female	Left	5	5	Seroma
16	74	Male	Right	8	10	Seroma
17	74	Male	Left	5	10	Seroma
18	76	Female	Right	6	8	Seroma
19	76	Female	Left	5	8	Seroma
20	65	Female	Right	6	5	None
21	65	Female	Left	5	5	None
22	65	Female	Right	8	4	Seroma

^**1**^*LOS* Length of Stay

The diagnosis was confirmed in all cases by pathological examination of the surgically excised specimens. Postoperatively, seromas developed in 8 lesions (36.4%), while a hematoma occurred in 1 lesion (4.5%). Seromas were managed with aspiration and compression bandaging without further complications, whereas the hematoma required surgical reintervention.

## Discussion

Elastofibroma dorsi is a rare, slow-growing, benign soft tissue tumor typically located under the scapula. According to the 2020 World Health Organization Classification of Soft Tissue and Bone Tumors, it belongs to the fibroblastic/myofibroblastic tumor group [13]. While its exact prevalence is not well known, a study of patients aged 60 and over who underwent CT scans found elastofibroma dorsi in 2% of cases [14]. The pathogenesis of elastofibroma is not well understood; however, histopathological studies suggest that it results from collagen fiber degeneration [15].

Patients report partial limitation and pain in shoulder function, as well as a hard, painless, tumoral swelling at the subscapular level when examined. Soft tissue ultrasound, computed tomography or magnetic resonance imaging is used to determine the lesions’ location, symmetric direction, and density patterns, as these imaging methods alone do not reveal specific features suggestive of elastofibroma dorsi.

Elastofibroma was located on the right side in 59.1% of our patients, on the left side in 40.9%. Similarly, in the study by Lococo and colleagues, the right side was more frequently affected (47.9%) [16]. More frequent use of the right upper extremity could be a predisposing factor. In the study by Parratt and colleagues, right-side involvement was also higher, at 53.3% [17]. Additionally, the researchers noted that nearly half of the tumors were located on the dominant extremity [17]. Based on lesion count, 15 lesions (68.2%) occurred in females and 7 lesions (31.8%) in males. Similarly, other surgical studies in the literature reported a higher proportion of female patients; for example, Deveci and colleagues reported a rate of 88.2% [18].

Observation is generally recommended for asymptomatic elastofibroma dorsi lesions smaller than 5 cm, whereas marginal resection is considered the optimal treatment for larger or symptomatic lesions [19]. In the present study, the mean lesion size was 7.0 cm (SD ± 1.41 cm), with a range of 5 to 10 cm, supporting the indication for surgical treatment in our cohort. Owing to the deep subscapular location of these tumors and the dead space inevitably created after excision, postoperative seroma formation has been reported as the most common complication, with rates ranging from 35.9% to 87.5% in previous studies [16, 18]. Consistent with the literature, seroma developed in 8 lesions (36.4%) in our series, while hematoma was observed in only one lesion (4.5%). Despite routine drain usage, postoperative fluid accumulation may still occur in this anatomical region. Importantly, all seromas were managed conservatively and did not result in major morbidity, infection, or persistent pain. Complications such as wound site infections and constant pain are also observed to a lesser extent in patients who undergo surgical treatment for elastofibroma dorsi [20].

The postoperative recurrence rate of elastofibroma dorsi is very low, as seen in the literature review. Nagamine and colleagues reported a recurrence rate of 1 case (0.58%), whereas Scamporlino and colleagues found no recurrence [21, 22]. Similarly, no recurrence was observed in our cohort during the follow-up period.

Although endoscopic approaches may theoretically be possible for the treatment of elastofibroma dorsi, the authors currently have no clinical experience with these techniques. In addition, given the lesion’s deep anatomical location and its typically fibrous structure, open marginal excision remains a simple, reliable, and cost-effective surgical method. Therefore, we believe that open marginal resection remains the most practical treatment option for symptomatic patients.

In the present series, six patients presented with bilateral involvement. Five of these patients were operated on in a single simultaneous surgical session, whereas one patient underwent a staged procedure. The choice between simultaneous and staged surgery was primarily guided by the patients’ clinical symptoms and preferences rather than by technical limitations. In the staged case, the patient initially presented with unilateral symptoms and subsequently requested excision of the contralateral lesion during follow-up after experiencing symptomatic progression. Importantly, no additional technical difficulty or intraoperative complication was encountered during the management of bilateral cases, regardless of whether surgery was performed simultaneous or in a staged manner.

Although elastofibroma dorsi has been previously described in the literature, studies reporting surgical outcomes from multidisciplinary collaboration between orthopedic and thoracic surgery teams remain limited. In addition, case series on surgically treated elastofibroma dorsi remain relatively uncommon due to the condition’s rarity. We believe that our series contributes to the current literature by presenting the clinical presentation, radiological characteristics, surgical management, and postoperative outcomes of patients treated in a multidisciplinary setting.

Our study has some limitations. The retrospective design and the relatively small number of cases may limit the generalizability of the findings. This is largely due to the rarity of elastofibroma dorsi and the limited number of patients requiring surgical treatment. In addition, standardized patient-reported outcome measures, such as the Visual Analog Scale (VAS), were not routinely recorded in the medical charts. Therefore, postoperative improvement was assessed based on routine outpatient follow-up and patient-reported symptom relief, reflecting daily clinical practice but limiting objective quantification of outcomes. Further prospective, multicenter studies with larger cohorts and standardized outcome measures may help to expand upon these findings.

## Conclusions

Elastofibroma dorsi should be considered in the differential diagnosis of subscapular soft tissue masses, especially in middle-aged women. Radiological imaging helps identify the lesion and delineate its margins. When imaging findings are atypical or suspicious, we recommend performing a preoperative Tru-Cut biopsy to exclude malignancy and avoid unnecessary surgery. Marginal resection can achieve curative results, and the placement of a drain with a compression dressing can minimize postoperative seroma and hematoma formation.

## Data Availability

The dataset generated and/or analyzed during the current study is available from the corresponding author.

## Abbreviations

CT: Computed Tomography
MR: MagneticResonance
CM: Centimeter
LOS: Length of Stay
